# Suggesting a mechanism for acupuncture as a global percutaneous needle fasciotomy that respects tensegrity principles for treating fibromyalgia

**DOI:** 10.3389/fmed.2022.952159

**Published:** 2023-01-27

**Authors:** Shiloh Plaut

**Affiliations:** Department of Primary Care and Population Health, University of Nicosia Medical School, Nicosia, Cyprus

**Keywords:** acupuncture, fascial armoring, fibromyalgia, mechanism, myofibroblast, needling, pain, treatment

## Abstract

Acupuncture is a minimally invasive therapeutic method that uses small caliber needles while inserting them through the skin into various areas of the body. Some empirical studies find evidence to support the use of acupuncture as a treatment for certain medical conditions, however, this peculiar practice is widely considered as the domain of alternative and non-evidence-based medicine. Several mechanisms have been suggested in an attempt to explain the therapeutic action of acupuncture, but the way in which acupuncture alleviates chronic non-cancer pain or psychosomatic and psychiatric disorders is not fully understood. A recent study suggested a theoretical model (coined “Fascial Armoring”) with a cellular pathway to help explain the pathogenesis of myofascial pain/fibromyalgia syndrome and functional psychosomatic syndromes. It proposes that these syndromes are a spectrum of a single medical entity that involves myofibroblasts with contractile activity in fascia and aberrant extracellular matrix (ECM) remodeling, which may lead to widespread mechanical tension and compression. This can help explain diverse psycho-somatic manifestations of fibromyalgia-like syndromes. Fascia is a continuous interconnected tissue network that extends throughout the body and has qualities of bio-tensegrity. Previous studies show that a mechanical action by needling induces soft tissue changes and lowers the shear modulus and stiffness in myofascial tissue. This hypothesis and theory paper offers a new mechanism for acupuncture therapy as a global percutaneous needle fasciotomy that respects tensegrity principles (tensegrity-based needling), in light of the theoretical model of “Fascial Armoring.” The translation of this model to other medical conditions carries potential to advance therapies. These days opioid overuse and over-prescription are ubiquitous, as well as chronic pain and suffering.

## 1. Introduction

Acupuncture is a traditional Chinese treatment method using fine needles that are inserted into various areas of the body. The origins of this strange practice date back several thousand years to the far East, with the canonical text established in the first century BC and known as the Inner Classic of the Yellow Emperor (1). Suggested mechanisms to explain acupuncture include neurohormonal pathways and induction or release of anti-nociceptive substances, activation of endogenous opioid mechanisms, stimulation of neuropeptide gene expression, the gate control theory, immunomodulation, reflex and spinal nerve stimulation followed by autonomic modulation, mechanical cellular signaling pathways, psychological or placebo effect, and “Qi energy” (1–5). While several explanations exist for its mode of action, acupuncture is often perceived as non-scientific. Until relatively recently, many in the west considered it the Chinese equivalent of voodoo (1). Despite the existence of some empirical evidence to support this therapeutic method for certain medical conditions, many physicians do not recommend acupuncture to their patients due to lack of a comprehensive theoretical model to explain it. Indeed, the treatment should be based on the pathophysiology of the disease. Often grouped with other alternative methods under one eastern umbrella, acupuncture stands out of the rest because it appears to be a minimally invasive surgical intervention of the fascia.

A recent study suggested a theoretical model (coined “Fascial Armoring”) with a cellular pathway to help explain the mechanism and pathogenesis of “functional somatic syndromes” (6). Syndromes such as “fibromyalgia,” “chronic fatigue syndrome,” “somatic symptoms disorder,” “myofascial pain syndrome,” and other psychosomatic/functional/“non-specific” conditions are suggested to be on a spectrum of a single medical entity (6–8). The underlying pathophysiology of these syndromes is suggested to involve, among several things, myofibroblasts in fascia that possess contractile activity with a positive feedback loop of force generation (6). This abnormality can lead to mechanical tension in the fascial tensegrity system and compression of anatomical structures, which can help explain the symptomatology and manifestations of fibromyalgia-like syndromes. Fibromyalgia is a chronic-widespread-pain-and-fatigue disorder with an unknown mechanism or cause, with much stigma. It affects 2–6 percent of the population (7) and is often managed by primary care physicians and rheumatologists. Current treatments are insufficient, causing frustration among patients and clinicians alike. No cure is known to exist. The opioid crisis in our society is ongoing, suffering is prevalent, and the social and economic burden of chronic pain conditions is high. In this paper the theoretical model of Fascial Armoring is presented in order to then discuss a new mechanism of action for acupuncture.

The following discussion is based on a literature review and aims to offer a new mechanism for acupuncture as a global percutaneous needle fasciotomy that respects tensegrity principles in light of the framework of Fascial Armoring. According to this suggested model, a decompressive mechanical treatment that applies multiple needles in different areas of the tensegrity system might be utilized to treat the fascial abnormality in myofascial pain/fibromyalgia syndrome and functional psychosomatic syndromes. Insertion of multiple needles, while letting the system completely rest, might allow internal fascial forces to tear fibers and release tensions in the system (6). It is a more yielding approach with less pain. In addition, compared to western dry needling (which elicits pain and may not properly treat the underlying disease), more compliance is expected from patients using this method. The holistic view of acupuncture aligns with the continuity and pervasiveness of fascia. In the long term, a method that respects tensegrity principles is expected to be much more effective as a treatment and prophylaxis of fibromyalgia-like diseases.

The key points setting the ground for this paper’s proposed hypothesis are as follows:

• Acupuncture is an intriguing ancient technique, considered by many as alternative medicine, and still lacks a clear mechanism to explain its practical application and theory.
• Fascia and proto/myo/fibroblasts phenotypes may have a major role in the pathophysiology of “non-specific” pain and psychosomatic syndromes.
• Fibromyalgia patients are found to have pathological changes in their extracellular matrix (ECM) and myofascial tissue.
• The theoretical model of Fascial Armoring integrates biomechanical properties of fascia and biotensegrity principles to propose a treatment for fibromyalgia-like conditions.
• A new mechanism of action for needling therapy was recently offered, whereby needling fascia relieves mechanical abnormalities in myofascial tissue and in the network of myo/fibroblasts, while taking into consideration the concept of tensegrity.

## 2. A brief explanation of the theoretical model of fascial armoring

In order to unfold the therapeutic action of acupuncture as a global percutaneous needle fasciotomy that respects tensegrity principles, we must first look at the model of fascial armoring and the process of myofibroblast force generation in the bio-tensegrity system of fascia. Fascial armoring is a theoretical model that was developed to help explain the pathogenesis of fibromyalgia-like syndromes (6). Functional psychosomatic syndromes are suggested to be on a spectrum of a single medical entity (7, 8), which may share a common rheuma-psycho-neurological mechanism (6). The fascial armoring model focused on the rheumatological facet of this medical entity. It suggests that these syndromes are driven, in part, by myofibroblast-generated-biotensegrity-tension, i.e., a network of myo/fibroblasts with contractile activity in fascia causing a connective tissue abnormality of the fascial biotensegrity system, leading to widespread mechanical compression (6).

The cellular pathway of the pathophysiology of fibromyalgia was suggested to involve mechano-signaling of myo/fibroblast phenotype cells in fascia (e.g., integrin and focal adhesion, Rho-associated-kinase, alpha smooth muscle actin, and transforming growth factor beta pathways) (6). Widespread mechanical tension and fascial rigidity might help explain several of fibromyalgia’s manifestations such as: pain, distribution of pain, decreased pressure pain threshold, chronic fatigue, morning stiffness, cardiovascular and metabolic abnormalities, autonomic abnormalities, small fiber neuropathy, close association with hypermobility syndrome, various somatic symptoms, absence of clear inflammation, silent imaging investigations, and other phenomena (e.g., occasional complete resolution soon after surgery) (6). The following findings are the basis for the theoretical model of fascial armoring: (i) Myofibroblasts can cause long-term contractures in tissue; they synthesize alpha-smooth-muscle-actin (α-SMA) fibers that allow them to lock in tension in the ECM in a slip and ratchet mechanism (9). Tomasek et al. described the complex biological and mechanosensitive activity of myofibroblasts and their stress shielding. Forces generated in ECM are maintained and reinforced over time by matrix remodeling and collagen deposition, accompanied by simultaneous myofibroblast contraction and force generation (9). (ii) Fascia forms a continuous network throughout the body and has qualities of tensegrity (or biotensegrity) (6, 10–12). Tensegrity is an architectural term describing how compressive and tensile element forces enable the dynamic stabilizing of one connected structure (10, 11). The term “biotensegrity” integrates complex biological aspects of living systems into a tensegrity biomechanical model where each “separate part” of the system is valued with relation to the whole (12). (iii) Fascia can transmit forces in the body to a distance with mechanical forces able to travel along myofascial chains (13). (iv) Fascia is able to actively contract (14). Myofibroblasts are present in fascia of normal individuals and can cause long term pathological contracture as shown in studies of Schleip and Klingler (15). (v) Soft tissue fibroblasts form a widespread interconnected cellular network with potentially critically overlooked physiological and functional importance (16).

These findings help form a theoretical model of fascial armoring- the creation of generalized tension and compression due to a widespread myofibroblast network in a stiff and rigid fascial system. Empirical evidence in support of the fascia-based pathogenesis of fibromyalgia, with widespread compression, can be found in studies such as:

1) A study by Katz et al. found significantly higher intramuscular pressures in trapezius muscle of fibromyalgia patients. The mean value of intramuscular pressure in the patient group was 33.48 mmHg compared to 12.23 mmHg in controls (17). The authors of the study stated that the burden of the pressure abnormality might contribute to diffuse muscle pain in fibromyalgia and may be an intrinsic feature of the disease. Therefore, fibromyalgia as a disease of exclusively central neuroplastic pain should be reconsidered (17).
2) Biopsies of fibromyalgia cases indicate definite and non-specific muscle changes which are suggested to be due to chronic muscle contraction and ischemia of unknown etiology (18).
3) Decreased peripheral blood flow in fibromyalgia patients was found, which suggests there are functional disturbances in their cardiovascular system (19).
4) In muscles of fibromyalgia patients, increased DNA fragmentation and ultrastructural changes are suggested to be secondary to chronic muscle contraction (20).
5) In a biopsy study of fibromyalgia patients, peripheral fibroblast transforming growth factor-β gene expression is significantly higher compared to controls (21).
6) The protein expression of genes involved in ECM turnover and oxidative metabolism has a differential expression in fibroblast of fibromyalgia patients, which could explain the inflammatory status of these patients (22).
7) A significantly lower amount of intramuscular collagen was found in fibromyalgia patients, which may cause a lower threshold for muscle micro-injury and result in non-specific signs of muscle pathology (23).

An elaboration and analysis with further empirical evidence that support fibromyalgia as an entity involving widespread mechanical compression can be found in a recent study (6). According to the model of fascial armoring, unhealthy lifestyle (e.g., sedentarism, western diet, obesity, and mechanical factors such as tight clothes) is suggested to be a major contributor to “functional-psycho/somatic syndromes” by stimulating fascial myofibroblasts. Based on the model, any factor that induces proto/myofibroblasts in fascia may increase risk for, or lead to, a fibromyalgia-like condition. It was hypothesized that a sever manifestation of fascial armoring might manifest as a mild-to-moderate-global-chronic-compartment-like syndrome, which may help explain various organic findings in fibromyalgia suggesting long-standing low-grade widespread ischemia (6).

## 3. Hypothesis

A treatment that uses multiple needles inserted in various areas of the body while respecting the complex properties of fascia is hypothesized to modulate the fascial bio-tensegrity system, thereby relieving mechanical tension in myofascial and subcutaneous tissue (6). I suggest acupuncture acts mechanically as a global percutaneous needle fasciotomy that respects the biotensegral properties of fascia (i.e., “tensegrity-based needling”) and can shift the state of the system to a more balanced state. Studies show that inserting dry needles has mechanical effects on fascia and alleviates pain (24–32). Modulating the bio-tensegrity structure and fascia may be a way to relieve mechanical pressure and treat the underlying fascial abnormality in fibromyalgia-like diseases (6). Tensegrity-based needling/acupuncture (needling while respecting qualities of tensegrity) is expected to lower the shear modulus of fascia over multiple sessions, and ultimately lead to significant effects on myofibroblasts in fascia through mechanosensitive signaling and changes in tissue stiffness (6). Fibromyalgia, even though currently defined as a central neuroplastic pain disorder, is dependent on peripheral input of nociception. Therefore, I reason that acupuncture might be able to treat the fascial abnormality of fibromyalgia-like diseases to help induce and sustain remission.

## 4. Methods

This work is based on a narrative review that used key phrases on fascia, myofascial pain, fibromyalgia, and acupuncture. The databases used were MEDLINE, EMBASE, COCHRANE, PEDro, and medRxiv. Systematic searches for keywords on fascia, fibromyalgia, acupuncture, and myofascial pain, were done between September 2020 and 6 February 2022. Non-systematic searches for keywords on related topics were done in PUBMED. Inclusion/exclusion based on title and abstract, then full text inspection. Items off topic and foreign language were excluded. A data charting form was used to abstract key data, as well as a summary document that was updated in an iterative process- that document evolved into this review. A total of 831 items were included in the literature of this study for the purpose of offering a new hypothesis and theory on acupuncture based on the model of fascial armoring.

## 5. Needling

Empirical studies suggest needling exerts mechanical effects on fascia and a mechanical action of needling itself relieves pain (24–26). Needling therapy lowers the shear modulus and stiffness of myofascial tissue (27–29). Langevin et al. show that the insertion of needles into fascia has important effects on surrounding tissue and cells (30–32). Fibroblasts contract upon release of tension in attached collagen lattices (33). Fibroblasts create tension in the attached matrix and when tension is released cells undergo contraction with a rapid contraction of the collagen matrix (9). It was suggested that needling may help relieve mechanical tension from the fascial fiber-cellular network and modulate the bio-tensegrity structure as a possible treatment for fibromyalgia (6). Evidence for the efficacy of needling techniques in relieving chronic pain and myofascial pain conditions can be found in literature (34–38). Two main types of mechanical needling therapy (without injection) are found in the literature which are: (i) western dry needling, and (ii) traditional Chinese medicine’s acupuncture. I would like to discuss these two methods in light of principles of the bio-tensegrity model of facial armoring, a framework called tensegrity-based needling (TBN) (6).

### 5.1. Dry needling

Dry needling is a relatively new minimally invasive method developed in modern medicine. It still lacks a comprehensive theory to fully explain its mode of action in myofascial and “non-specific” or psychosomatic diseases, mainly because these conditions lack a clear pathophysiology and mechanism themselves. Ideally, any treatment method should theoretically conform to the pathophysiology of the disease that it aims to treat. Some empirical studies support western dry needling, although most of the studies examine its short/medium-term effects, whereas the long-term effects are still unclear (36, 39–41).

Western dry needling can be seen as a variation of trigger point injection (without the injection) or an uncomplicated variation of acupuncture. Used often to treat myofascial pain and trigger/tender points, dry needling can be extremely painful for the patient (29), some might even call it abusive. In dry needling for myofascial trigger-points, a needle is often inserted forcefully and repeatedly deep into the trigger/tender point during a session (e.g., fast-in and fast-out/pistoning/sparrow pecking maneuver, possibly in a fan or cone shape), with the purpose of “destroying” the trigger point or trying to induce edema, positive inflammation, and self-healing processes; or aiming to relieve the pain through spinal cord afferent signaling or other mechanisms (42–45). However, as many clinicians’ experiences might indicate, these mysterious painful points recur a short while after treatment too often, if not in the same site, then in a different one. Interestingly, sometimes pain migrates.

Many patients are deterred from repeating this type of treatment due to extreme pain elicited by needling (called “post-needling soreness”) (46). If the trigger point recurs, either in the same spot or a different one, the needling procedure can be repeated. However, in cases where the patient’s pain eventually becomes diffuse and widespread, the underlying diagnosis can be altered to a psychologically driven pain or “chronic widespread pain”/“fibromyalgia,” for which the treatment nowadays is often psychological or psychiatric. Cognitive behavioral therapy is one such modality for diffuse pain (47). Noteworthy, chronic widespread pain is not considered by some certified clinicians as a real organic medical condition to treat (48). Fibromyalgia is one example of such a prevalently neglected and stigmatized medical condition (where many certified clinicians actually think that the patient is imagining the pain).

### 5.2. Acupuncture

Acupuncture is an important therapeutic method in East Asian medicine. Its theoretical and philosophical framework differs from that of the modern West. East Asian medicine tries to formulate a qualitative diagnosis in the overall entirety of a person’s state, signs, and behaviors. The west’s biomedical approach aspires to achieve a scientific and measurable quantitative assessment of an organic biological process (1). Eastern philosophy, such as yin-yang and Qi, is integrated into acupuncture and is one of the reasons for the difficulty of accepting it in western thought and science (1). Moreover, acupuncture uses a network like view of the human body with channels of flow of energy that can be needled in certain points to correct an imbalance or to modulate internal elemental life energies. Multiple needles are gently inserted in various area of the body, sometimes in a distance from the chief area of complaint. The scientific measurable justification for the theory and philosophy behind acupuncture is not an issue for traditional eastern thought and practitioners (1).

Evidence supporting the use of acupuncture in chronic pain and “psychosomatic” conditions can be found in literature (49–53). The mechanism of acupuncture is not fully understood, and many theories have been proposed to explain it, e.g., neurohormonal pathways and induction or release of anti-nociceptive substances, activation of endogenous opioid mechanisms, stimulation of neuropeptide gene expression, immunomodulation, the gate control theory, mechanical cellular signaling pathways, Qi energy, psychological effect, placebo effect, and so forth (1–5).

Overall, acupuncture is considered safe when performed by a competent trained practitioner (1, 54, 55). In rare circumstances, acupuncture can cause complications such as those associated with any other type of needle use (dizziness, minor hemorrhage, pain, infection, or more serious events such as pneumothorax) (1, 56–58). Adverse events usually occur when the acupuncture practitioner has inadequate training (1).

### 5.3. Tensegrity-based needling

Tensegrity-based needling (TBN) was suggested as a treatment method based on the facial armoring model of myofascial pain/fibromyalgia syndrome (6). It utilizes a tensegrity network conceptual framework to treat “functional psycho/somatic” or “non-specific” pain conditions. Tensegrity states that structures (or tensegrity systems) are stabilized by continuous tension with discontinuous compression, and function as one connected system (10–12). TBN suggests that needling therapy should take into consideration the bio-tensegrity qualities of fascia and its interconnectedness and continuity throughout the body. Needles are inserted in different areas of the system in order to lower global fascial stiffness and aim to collapse the tension of the system in a synchronized and directed manner (6).

The forces imposed in muscles are distributed over the tissue as a whole and transmitted by intramuscular connective tissue (59). Skeletal muscles are linked by fascial tissue and form a body-wide network of multidirectional myofascial continuity (60). A 2004 study suggests that soft tissue fibroblasts form an extensively interconnected cellular network, suggesting they may have important overlooked physiological functions (16). Fascia has contractile abilities and contains contractile cells (proto/myofibroblasts) (14, 15). A fascial myo/fibroblast network was suggested to cause long term contracture and tensegrity tension in fascia; therefore, needle insertion throughout this network is expected to cause mechanical disruption of the ECM’s fiber-cellular network and modulate abnormal tensions in the fascial system (6).

TBN is based on three elements of fascial armoring. These are (i) tensegrity mindset in the continuity of the fascial system (ii) trigger points and satellites (iii) a proto/myo/fibroblast population (network) creating tension, contracture, and stress shielding.

The mechanical action of needling has physiological and therapeutic importance: studies suggest the needle insertion itself relieves pain (25, 26, 37), which might suggest needling creates a focus for a mechanical tearing action on fascia (6). Needling exerts mechanical effects on tissues (30–32). Under the framework of fascial armoring, the tension and shear stress inside connective tissue pull on the point of fine needle insertion until the sum force vectors (or horizonal components) at that point is eliminated or needle is removed (6). Eliminating tensional forces and cutting off fascial fibers causes cells to rapidly contract and subsequently lose their stress fibers and adhesion complexes (9). Myofascial trigger points and satellites were suggested to be manifestations of myofibroblast populations in fascia with mechanical tensegrity links (6). Myo/fibroblasts create tension in the attached matrix; when tension is released cells undergo contraction with rapid contraction of the ECM (9). Studies observe changes in contractility and stiffness in fascia, including “needle grasp” phenomenon when needles are inserted (30–32). Needle delivery systems deform and move soft tissue and organs (61, 62). These mechanical effects may suggest that inserting a needle causes windup of connective tissue and modulation of the fascial system’s fiber-cellular network, guided by its internal forces and contractile cells. Studies suggest needling affects facia both at the insertion point and further at a distance (3, 32, 61, 63). Damage to fascia inflicted by a needle might actually allow for eliminating tensions between tense nodes in the network of (myofibroblasts) myofascial trigger-points and satellites. If inserted deep enough, it might also free edematous fluid, hyaluronic acid, and other factors trapped within the layers (e.g., serotonin). Micro-dialysis studies of trapezius muscle show myofascial pain syndrome patients have increased concentration of nociceptive and inflammatory substances in myofascial tissue (e.g., substance P, interleukins, tumor necrosis factor alpha, etc.) (64–66), and fibromyalgia patients have higher levels of lactate and pyruvate in myofascial tissue (67). Relieving mechanical tension might improve prefusion and lymphatic drainage, leading to better clearance of nociceptive and inflammatory substances (6). While one study suggests needling can improve blood perfusion (68), another study showed infiltration of trigger points can improve symptoms of intermittent claudication (69).

In short, TBN is expected to gently change the dynamics of the tensegrity structure and shift the state of the system to a more balanced state. The theoretical end purpose for this treatment would be to progressively collapse the tension of the system in a concentric way throughout multiple sessions by passively inserting multiple needles and then allowing the network of cells to lower global fascial stiffness beneath the threshold necessary for myofibroblasts activity (∼10–20 kPa) (6). One study suggested tissue stiffness of ∼20 kPa is the threshold for robust myofibroblast α-SMA synthesis (70). Upon complete freeing of tension in the attached matrix, myofibroblasts undergo apoptosis with reduction of α-SMA (71). Each intrafascial/subcutaneous needling treatment should be adapted to the current state of the bio-tensegrity structure. Ignoring tensegrity principles is expected to lead to more pain, either in the immediate, short, or long term. As an example, to illustrate this model, limb amputation is expected to lead to high risk of chronic pain due to a new imbalance in the fascial system (6). Studies suggest needling lowers myofascial tissue stiffness as measured by shear wave elastography (27–29). Releasing tensions in ECM might also relieve stretch lesions along sensory/sympathetic nerves (6). The more stress-shielding is present, the longer it should take until significant symptomatic improvement is achieved. Under this framework, needling therapy actively breaks the connections in the tensegrity-like network of fascia and then allows for new healthier connections to form *via* natural remodeling of the ECM (6). After modulation of the tensegrity structure, fascia will regenerate *via* myo/fibroblasts. Studies show fascia regenerates within several months after fasciectomy/fasciotomy (72, 73).

**FIGURE 1:**
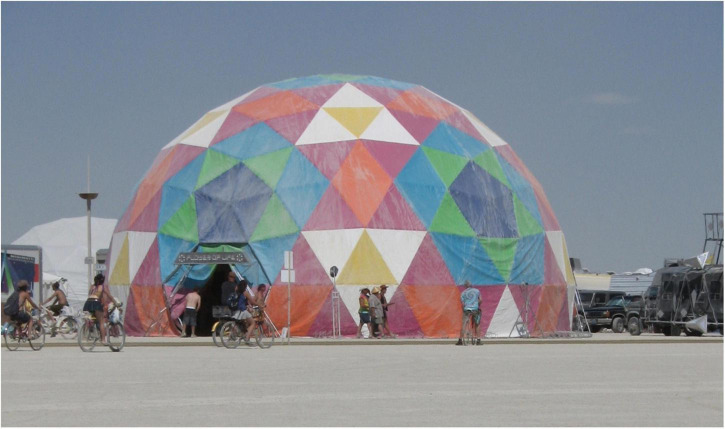
A geodesic model of the bio-tensegrity structure. Each sheet may represent an area of fascia in the system. Each “sheet” has its spring constant set by fascial qualities and myofibroblast generated forces. Mechanical forces can travel to other areas through connections in the structure and myofascial chains. With permission from PACIFIC DOMES Inc. www.eventdome.wordpress.com. Reprinted from https://eventdome.files.wordpress.com/2010/07/bm-multi-colored.jpg, with permission from Sequoia Miller from Pacific Domes Inc.

**FIGURE 2:**
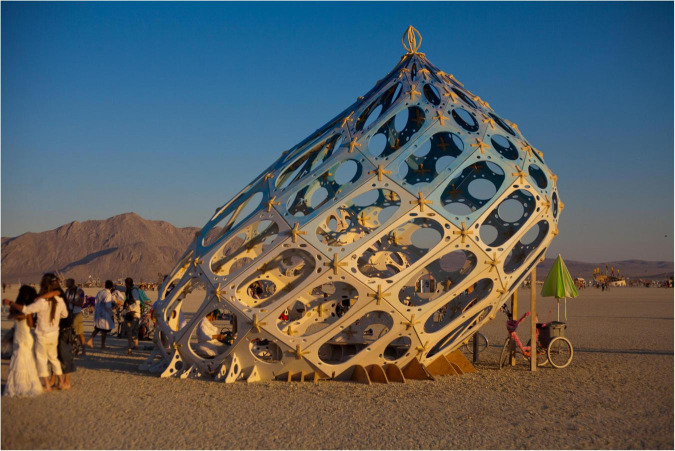
A bio-tensegrity structure with imbalance. Nerves can be affected by internal fascial forces and sense tension to send pain signals. This happens more in weaker areas of the structure, i.e., areas that are pulled by myofascial tissue and myofibroblast generated forces, for example, as seen on the right side of the structure when the dome is facing the observer. Needling the painful area does not address the underlying cause for the pain, which may be increased tension generated by the left side of the structure (when facing the observer). To balance the structure, one should needle the contralateral side to pain as well: the side to which the structure leans and which indicates more stress shielding and force generation. In this model, pain and somatic manifestations are a symptom of imbalance rather that the problem to simply target anatomically. With permission from, and photographed by, Aaron Neilson-Belman AaronNeilsonBelman.com. Reprinted from https://hippievanman.com/photos (accessed 2021).

Under the framework of tensegrity and fascial armoring, one might consider first needling the dorsal foot and foreleg in plantar fasciitis, for example, if connective tissue is pulling the plantar fascia and triggering pain. Thus, aiming to treat the underlying tensional abnormality in the connective tissue, and not simply the symptoms of pain. Importantly, needling the foreleg would change other areas of the tensegrity system, because tensegrity domes are made of many nodes- and changing one node changes the dome. Figures 1, 2 depict the framework of the fascial armoring model as a geodesic dome to illustrate fascia as a complex connected system susceptible to imbalance.

Realizing the tensegrity qualities of fascia, it seems that a trigger point with severe localized pain may not necessarily be the source of pathological tension because myofibroblasts stress shield their area from tension. Targeting only the symptom of pain during treatment is expected to lead to an endless chase of migrating pain whose location is determined by dynamics of the bio-tensegrity system (6). Tension may have a tendency of developing near hard and angled surfaces with sharp force-gradients. Tension and myofibroblasts in connective tissue will be present, but pain depends on the degree of sensory nerve involvement (6). Developing a therapeutic method that treats such a complex dynamic affair is not a trivial matter.

## 6. Discussion

In 1815 Dr. William Balfour described his method of “curing” patients with “rheumatism” (i.e., fibromyalgia) (74). Balfour applied bandages to treat patients with fibromyalgia or chronic “non-specific” pain at the area of complaint and saw immediate improvement in pain, joint range of motion, and ability to move. However, some patients returned a short while later with pain that migrated to a different area. The “cure,” as it seems, was only symptomatic and temporary (e.g., the case of Mrs. M. pp. 179–180) (74). Mechanical signals feed input into the myofibroblast positive feedback cascade of transforming growth factor beta-1 and α-SMA synthesis, which can lead to further contraction and tension in the ECM in a detrimental loop (9). This suggests that Balfour’s method modulates the tensegrity dynamics but in an opposite way compared to needling and acupuncture (6). Bandages (as well as taping and compression techniques) can modulate the tensegrity structure and/or induce myofibroblasts, matrix remodeling, and stress shielding, but fail to resolve the overall tension. TBN, on the other hand, is expected to slowly release the tensions from the tensegrity system, lower tissue stiffness, and, over time, treat the patient’s underlying fascial tensegrity abnormality. TBN, when done correctly, is expected to effectively treat- and perhaps even completely relieve- the underlying disease, according to the fascial armoring model.

Many controlled trials of acupuncture in the research literature are described as lacking strong evidence (38, 53). Noteworthy, establishing a proper control group is difficult in such studies. Finding a suitable placebo is somewhat challenging, and as it seems, any sham needling is not inert with regards to tensegrity dynamics. Moreover, some studies adopted a western non-holistic approach while using a fixed, drug-like, well-defined study protocol of needling a single acupuncture point for a particular symptom, even though this approach has little relevance to the methods of an acupuncture practitioner in clinic (1).

The terminology regarding dry needling in literature can benefit from being more consistent. In this paper, the term “dry needling” refers to any technique that suggests one should insert needles into points/areas of pain as the only strategy of the therapy, without embracing a holistic view of the body or the bio-tensegrity system. This encompasses “intramuscular manual therapy” or “trigger point dry needling” and any other technique that sees “non-specific” pain as the problem to target anatomically rather than a symptom of imbalance in a complex fiber-cellular network of fascia. A guiding principle of TBN is that imbalance in fascia should be balanced by modulating multiple areas of the tensegrity system simultaneously (*via* mechanical needling) to minimize any exacerbation and carefully shift the imbalance of the system toward a more balanced state. Geodesic tensegrity domes are made of many nodes- and to change a node is to change the dome. In this discussion, a geodesic tensegrity dome is a parable for the (fascio)musculoskeletal system, for the purpose of simplicity. As biology does not separate or segregate itself into different medical specialties, an imbalanced tensegrity structure is likely to be associated with an imbalanced mind (6). The “body” and the “mind” are one Being, one flesh.

### 6.1. Acupuncture as tensegrity-based needling (TBN)

**FIGURE 3:**
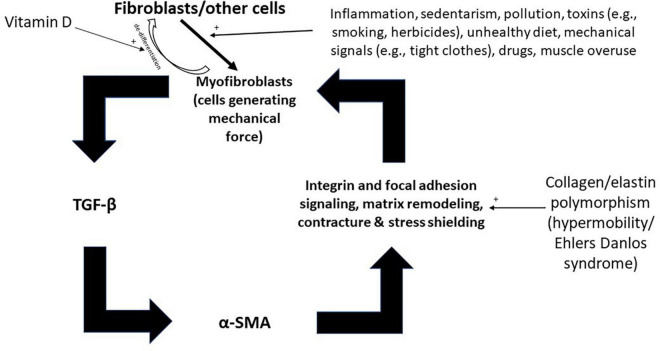
Inputs into the positive feedback loop of fascial armoring (fascial rigidity and biotensegrity tension). Fibroblasts, adipocytes, and other cell-types can differentiate to myofibroblasts. Empirical studies show various factors influence fibroblast-to-myofibroblast differentiation and α-SMA synthesis, such as: immobility, infection/inflammation, diet, herbicides, drugs, epi/genetics, etc. (6). Once myofibroblasts activity is initiated, a positive feedback loop can stimulate and maintain fascial armoring (i.e., lead to a chronic fascial bio-tensegrity pathology driven by a myo/fibroblast network of contracting cells in connective tissue). Open arrows with a plus sign indicate upregulation/stimulation. TGF-β, transforming growth factor beta; α-SMA, alpha smooth muscle actin.

Studies find needling lowers shear wave modulus of myofascial tissue (29). Systematic reviews and meta-analyses find acupuncture alleviates pain and stiffness in fibromyalgia and in myofascial pain syndrome (34, 36, 38). The proposition that acupuncture may be a form of TBN is derived from the marked similarity between the two methods and the holistic view of the body they both embrace. The following points suggest acupuncture may function as TBN:

(1) In traditional eastern acupuncture, several needles are often used simultaneously and in multiple areas of the body, as recommended by TBN. Between five and fifteen needles are typically used in acupuncture treatment, with the point combinations varying over the course of the sessions (1).
(2) Acupuncture points adhere to symmetry of the human body. Symmetry during needling reflects the understanding that affecting one side of the tensegrity structure can affect the reciprocal contralateral side of the structure, and a modulation of one side alone can exacerbate any pre-existing imbalance. Inserting a needle into one point on the midline respects right-left symmetry but not anterior-posterior or cranio-caudal chains of force transmission. Needling bilateral points off the midline respects left-right symmetry. The skeleton is mostly symmetrical across the mid-sagittal plane, but internal organs and their fascia are not necessarily so.
(3) Needles are often inserted into a distant site further away from the chief area of complaint (75, 76), or sometimes in the contralateral side (77). This is a guiding principle in TBN. Since myofibroblasts stress shield themselves and remodel ECM, a painful point is not necessarily the source of pathological tension but rather may be a sign of a weak area that is being pulled by more distant myofascial tissue (*via* myofascial or biotensegral channels or chains). In acupuncture therapy for plantar fasciitis, for example, needles are often applied in the dorsal side of the foot and in the foreleg (points designated BL 57, BL 60, ST 36, SP 5, etc.) (78, 79). According to TBN, the foreleg and dorsal side of the foot, as well as more proximal points in the leg, are reciprocal areas that should be needled because they act to pull the plantar fascia and cause tension and pain. A study indicated that tightness in the gastrocnemius and hamstrings is associated with plantar fasciitis (80, 81). In lateral epicondylitis, points in the wrist and the elbow are needled, including in the anterior surface of the upper limb (82). In “neuropathic/neuroplastic” limb pain, needles are inserted into the contralateral limb to relieve pain (77). In tension-type headache, adjacent points can be used, bilaterally, including the adjacent large myofascial tissue of the trapezius and sternocleidomastoid (83). In acupuncture theory, headaches can be treated by placing pins in the hands (1). The rationale for this, even though may sound peculiar to a reader who is not familiar with the concept of tensegrity, is that the myofascial tissue in the upper limbs is suggested to pull the fascia of the head and transmit the tension through myofascial chains and tensegrity connections. Fascia is continuous from the trunk across the upper and lower limbs, thus capable of affecting range of motion. Fascia connects the ankle and hip not only anatomically, but also mechanically (84). While one study demonstrated existence of an anatomical continuity between all muscles of the flexor region of the upper limb (85), there is evidence in support of direct serial tissue continuity from the neck and shoulder area to the forearm (86). Reportedly, an anatomical myofascial continuum exists between the neck, head, and eyes (87). Most human skeletal muscles are directly linked by fascia and connective tissue (13).
(4) Acupuncture points are distributed in a higher density in areas of bone protrusions (88) and are distributed in a systemic network-like relationship of channels (88–90). Needling points adjacent to bone protrusions and solid curved surfaces is a general principle of TBN because those are the areas where tension and/or myofibroblasts tend to develop, even if asymptomatic or subclinical (6). Solid surfaces cause more pressure and stronger mechanical signals when fascia is chronically pressed against them in a person subjected to factors such as sedentary behavior and tight clothing. Chronic mechanical stimuli then lead to induction of myofibroblasts and their cascade of contracture. The development of myofibroblasts and fascial armoring will also depend on the type of tissue and its mass at the involved area. Mapping areas and points adjacent to hard surfaces and myofascial tissue, while avoiding important structures such as nerves and blood vessels, while also considering myofascial chains, might correlate to a certain degree with the distribution and relationship of acupuncture points. A study aimed to evaluate the correlation between trigger points and acupuncture points for pain (91). It found a high degree of correspondence (approximately 70 percent) between these two sets of points. The close correlation suggests that trigger points and acupuncture points for pain represent the same phenomenon and may be explained in terms of the same underlying mechanisms (91). Non-trigger-point needling might influence muscular and neurovascular structures as well as the connective tissue. Noteworthy, trigger points and acupuncture points should not be expected to correspond entirely under this framework, because of the complexity of the tensegrity system. Tension developing in one area will often have a reciprocal area to be needled (even if not painful). For example, needling the area of the mastoid process (bilaterally) might require needling the back, the legs, or the middle of the forehead in the future even if the forehead is mostly flat, and even if the forehead does not have a painful trigger point. This is because releasing tension from the back of the head can shift imbalance to the front of the head, especially in a person with myopic vision overuse or a person overusing myopic vision during the needling session. The topography of acupuncture points might reflect tensegrity tension and its dynamics, and not simply myofascial painful points. Pain is a symptom of the entity; it is not the entity. Worth noting, the area of the abdomen does not contain many bone protrusions (although the anterior superior iliac spine should not be overlooked), however, bloating and sedentary behavior, as well as poor diet, belts and tight clothes, might lead to fibroblast-to-myofibroblast differentiation (with downstream physiological consequences). Fibrosis occurs in immobilized contracted muscles if they are maintained in the flexed position (59, 92). Sitting maintains abdominal muscles in the more flexed position. Psychological stress can potentially increase chronic abdominal muscle tone, thereby exacerbating the issue. Integrating the enteric nervous system and gastrointestinal tract complicates this model, though, overall, bloating acts to feed mechanical stimuli into the myofibroblast cascade of this model. Figure 3 shows the vicious cycle of fascial armoring and factors that modulate the myofibroblast cycle of force generation and tissue contracture (Figure 3). Poor diet can have biochemical effects and effects on the microbiome to induce myofibroblasts (93–95). Needling the abdominal fascia or Linea alba might require reciprocal needling in all four limbs and head to maintain balance in the fascial system.

(5) In acupuncture, needles are inserted once in a session, and patients are allowed to fully relax and rest. Manipulation of a needle once inserted is performed momentarily and minimally, only if necessary. Needle twisting may be utilized to induce a mechanical response by myofibroblast contraction around the needle. Twisting the needle winds up ECM and fascia around the needle causing increased tension momentarily. Myofibroblasts have stretch activated calcium channels, and intracellular calcium and myofibroblast contractility are mechanistically linked (6, 96, 97). The direction of twisting can increase or decrease tension in the fascia, depending on the forces in it and the direction of twist. In certain cases, twisting the needle after insertion can help release tensions, for example, in cases where fascia has become very fibrotic with much remodeling and stress shielding. If a torque force exists in an intertwined fascia, twisting might help relieve it. Nonetheless, increasing tension *via* twisting in specific points will modulate the tensegrity forces around that area (and along that chain), and then, the needle will tear fibers throughout the session, as explained in the findings section. Encountering microscopic blood vessels/capillaries throughout the tearing process of fascia might lead to minor bleeding by the end of the session. The more bleeding and erythema, the stronger indication it is that much tearing of fascia occurred (assuming a blood vessel was not accidentally punctured). Too much bleeding is a sign of unconstrained modulation of fascia.
(6) Pain is something to avoid, not induce. As is seems, the pain and post-needling soreness of western dry needling is not a complication of the technique but rather it is a direct result. An important principle of TBN which is adhered to by acupuncture is allowing the myofascial system to rest during treatment. Elicited pain and the accompanied muscle contractions of patients during a “dry needling pistoning maneuver” is absolutely avoided. Rest and relaxation are of special importance during treatment. The fascio-musculo-skeletal system needs to be relaxed during treatment according to TBN, as well as the relaxation of the mind. When there is rest and relaxation, the internal fascial forces guide the process of fascial tensional release, which is a key aspect of TBN.
(7) The order of sequential needle insertion is a factor that is given attention in acupuncture (98). In TBN, if imbalance in the tensegrity structure is detected in the right upper part of the body, for example, it might require needling the right lower limb, left lower limb, and left upper limb, working around the structure to collapse the tension in a controlled fashion. If, for example, imbalance and pain is detected in the lower limb (e.g., following below knee amputation), needling might be done in the head, unilateral upper limb, contralateral upper limb, and contralateral lower limb, while working around the weak area of the structure. The back and thoracolumbar fascia should be needled to help relieve tension traveling down the lower limb. Determining the order of needling is complex under this framework and requires further understanding. Much research is required before translating this oversimplified discussion into clinical practice.
(8) Systemic symptoms require needling in multiple points throughout the body (98, 99). According to fascial armoring, functional-somatic or non-specific symptoms such as fatigue can be a sign of widespread fascial compression or a global chronic exertional compartment-like syndrome. Fascia is an interconnected fiber-cellular network like a woven net, with myofibroblast nodes/populations that create tension, and trigger points and satellites (6). Tension in a woven net cannot be released by cutting one string.

Even if a series of needling treatments does not induce complete relaxation in the tensegrity structure, it most likely makes the body more receptive to relaxation because it decreases mechanical tension and sympathetic tone. Sympathetic tone can be generated by pain and by fascial forces or stretch lesions in the nerves embedded in fascia (6). The bio-tensegrity structure is dynamic and each patient’s structure and the imbalance in it is unique to that individual at that point in time. Therefore, each needling treatment should be adapted according to the current state of the patient’s tensegrity status and modified according to the response to treatment. In other words, one needling protocol does not fit all, and does not fit one at all times. A reader who is familiar with acupuncture probably understood this concept already. Clinical examination is important and should be done prior to each needling session. Developing a method to monitor the state of the body’s fascial tensegrity structure by palpation would be an astounding achievement for modern medicine. The purpose would be to palpate and monitor the fascial system and to feel its qualities, not the cardiac pulse. Although, a weak string like cardiac radial pulse might be a marker for a widespread compression by an abnormal bio-tensegrity structure. A disease that is an “exertional chronic compartment-like syndrome of the whole body” should be easily felt and evaluated *via* palpation by a skillful individual (if one is aware of what to look for). Placing only two fingers on the wrist might not be sufficient to detect lateral movement or changes of the fascia beneath the skin during palpation.

Non-ideal modulation of the tensegrity structure, resulting from needling in a wrong way, would lead to exacerbation of pain and symptoms, either immediately or in the long-term. The therapeutic process should be gradual and progressive spanned over multiple sessions because changing the tensegrity structure too quickly may cause serious harm (6). Some suggest needles should remain for 20–30 min during each session, with a frequency of approximately two sessions per week (83). Fascia might have enough hidden potential elastic energy to cause bone fracture (6). A sudden change in fascia might shift tensegrity forces and exacerbate an imbalance (100).

Choice of needle depth: the precise depth of needling is an important variable. De-qi (needle grasp) describes a successful needling (31). It is a mechanical phenomenon. It may actually be the phenomenological representation of myo/fibroblast rapid contractions in connective-tissue (31) which is mediated by smooth muscle actin and calcium-myosin mediated contraction (6). Myo/fibroblasts have stretch activated calcium channels, and intracellular calcium and myo/fibroblast contractility are mechanistically linked (96, 97). Needle insertion can induce temporary contraction and increase the local tension. Following this increase in tension, if let rest, the needle will tear fascial fibers and slowly release tension (6). To “puncture the bone without damaging the tendons, to puncture the tendons without damaging the muscles, to puncture the muscle without damaging the “pulse,” and to puncture the “pulse” without damaging the skin” (Huangdi Neijing Suwen), seems to suggest that a therapist should be able to target and modulate the tensegrity system with caution and precision (the term “pulse” refers to fascia under this framework). If the needle does not penetrate the proper layer, the tensegrity structure will not be able to release its internal tensions in a healthy manner. If inserted too deep, it may cause harm. The angle of insertion affects depth. Worth noting, the repeated action of needle penetration in dry needling is neither very careful nor delicate and should be avoided, according to this theory. The long-term consequences of dry needling while ignoring tensegrity dynamics are not entirely known.

### 6.2. “Meridians”: A bio-tensegrity map?

The idea of meridians and their mechanical significance is interesting under this framework. It may be possible that the fascial tensegrity structure tends to have a conserved topography of tensional forces. Variations in this topography may also exist. Figure 2 depicts a tensegrity structure with imbalance whereby imbalance in the structure can manifest in different anatomical areas due to myofascial forces traveling in the system. Any specific imbalance in the fascial system would require needling in reciprocal areas. Imbalance or asymmetry in the tensegrity structure might have a tendency to reach certain energetic states which are stabilized by abnormal myofascial tensegrity forces. Also, the specific state of the tensegrity structure will be affected by elements of the musculoskeletal system. The skeleton is somewhat conserved between individuals but still has variations. Also, hypertrophy of a certain muscle group can cause imbalance in the structure. If, hypothetically, meridians serve as a topographical guide for the location of needling that can alleviate compression and shift the tensegrity dynamics to a more balanced state, it will further support the notion that acupuncture acts as TBN. If meridians correspond (to a degree) with myofascial/tensegrity chains, acupuncture as TBN would make even more sense. Any therapy that modulates the bio-tensegrity structure would have to take fascia’s complex dynamics into consideration while understanding that forces can be transmitted to a distance *via* myofascial chains. Myofibroblasts can be arranged in nodes or in lines, and lines of force are found to exist in tissue (101–106). For example, cardiac myofibroblasts tend to align their shapes and forces with the long axis of a wavy substrate (107). Myofibroblasts reorient themselves toward the direction of a stretch force exerted on them (108). Substrate concavity and convexity also affect mechanics of cells (109).

The ability of tensegrity forces to flow freely throughout this fiber-cellular network and in the system of fascia (or the “pulse”) might be related to the idea of a palpated “Qi energy.” This notion is presented assuming traditional Chinese philosophy accepts that even the metaphysical can have a physical representation in the body. If mechanical needling affects a person’s “Qi,” then “Qi” should have a mechanical aspect in the body. The Body-Mind-Spirit is one Being. This discussion is not aimed to validate or refute the idea of Qi energy or the whole philosophical background of acupuncture.

### 6.3. Acupuncture for fibromyalgia: Should we expect relapses until remission?

Since studies suggest fibromyalgia is overall dependent on peripheral input (110), interventions such as TBN/acupuncture aiming to target peripheral tissue have a role for central components of fibromyalgia as well as for treating their fascial abnormality. Allodynia and hyperalgesia in fibromyalgia can be improved or abolished by removal of peripheral impulse input (110). Mechanical hyperalgesia in fibromyalgia patients is likely a consequence of abnormal input from sensitized peripheral tissue receptors and resultant central sensitization. Abnormally sustained impulse input from muscle receptors in fibromyalgia patients and/or muscular ischemia appear to be a relevant mechanism for chronic muscle pain in such patients (110). Research has yet to establish if enhanced responses to somatic and cutaneous stimuli in fibromyalgia result from facilitating mechanisms within the brain, spinal sensitization maintained by tonic impulse input from somatic tissues, or abnormal mechanisms of descending facilitation from the brain to the spinal cord and/or somatic tissues (110). Nevertheless, recognizing the elevated intramuscular pressure as a possible mechanism for hypoperfusion and subsequent diffuse muscle pain in fibromyalgia suggests a therapeutic role for reducing intramuscular pressure as an intervention (17).

In the fascial armoring model, hyperalgesia would likely be affected by the state of nociceptors in abnormal fascia. Chronic nociceptor and mechanoreceptor activation, mechanical compression of neural structures, increased circulating inflammatory signals, and cytokine induced peripheral sensitization, are some of the pathways possibly leading to long term changes in the brain of these patients. Relieving a fascial biotensegrity abnormality and its noxious stimuli may be relevant for neurobiology by affecting input to the central nervous system including the somatosensory cortex, amygdala, brainstem, and insular cortex, reducing physical-emotional stress, affecting pathways of peripheral and central sensitization (including spinal and dorsal root ganglion), reduce microglia activation, and conditioned pain modulation, among many other related processes and paradigms neuroscience is familiar with. Treating their fascia properly is also expected to lead to less “pain catastrophizing” and should be more therapeutically effective than, for example, acceptance and commitment therapy.

Clinical trials show a beneficial effect of acupuncture for fibromyalgia (34, 38). According to the fascial armoring model, relapse is expected to happen after treatment due to the proto/myofibroblasts that remain in fascia (6). Myofibroblasts contract and lock in tension in the ECM over several hours (9). Relapses might occur a short while after each treatment. This will happen until global tension is eliminated or decreased enough to downregulate α-SMA and cause myofibroblast de-differentiation or apoptosis (6). A randomized controlled trial studied dry needling and extracorporeal shockwave therapy and measured their effect on the stiffness and shear modulus of fascia using shear wave elastography (27). Results showed both modalities significantly lowered the stiffness of fascia as measured by shear wave elastography. The shear modulus measured 1 month after treatment was significantly lower than pre-treatment values, and these values corresponded with lower patient reported pain and pressure pain thresholds. However, when measured at 3 months post-treatment, the shear modulus had increased above 1-month post-treatment values. Stiffness seemed to recover to a degree, perhaps reflecting the start of recurrence. The authors noted that the mechanisms of elastic recovery after the extracorporeal shockwave therapy and dry needling treatment are unclear (27).

According to fascial armoring, as long as proto/myofibroblasts are present in fascia, and as long as lifestyle continues to feed input into the cascade of fascial armoring, relapse of tension, stiffness, pain, and increased shear wave measurements is expected to occur due to matrix remodeling and myofibroblast force generation. Based on fascial armoring, TBN therapy (and any needling therapy) initiates a process of tensegrity modulation that should be then supported and continued by the patient following treatment. Release of tension from of the bio-tensegrity structure is a dynamic ongoing process. Therefore, movement, physical exercise, maintaining proper posture, and relaxation techniques are required especially during the period following each needling session and as a lifestyle intervention.

### 6.4. Suggesting a role for acupuncture in the prevention of chronic widespread pain and psychosomatic disorders

The dynamic nature of ECM synthesis as an ongoing process suggests that homeostasis and balance normally exist between matrix synthesis and degradation (remodeling), between myofibroblast induction and de-differentiation, and between cellular force generation and tensional release. It is a delicate interplay of multiple factors (e.g., collagen, elastin, matrix metalloproteinases, growth factors, etc.). If any imbalance develops in the fascial bio-tensegrity structure, it can help maintain and propel the myofibroblast cascade of contracture *via* mechanosensitive signals, therefore driving the positive feedback loop of myofibroblast contractions and stress shielding (6). Studies show a close association between hypermobility/Ehlers-Danlos syndrome and fibromyalgia/psychosomatic disease (111–114). Chronic widespread pain, fibromyalgia, and hypermobility syndrome pain, share common features and perhaps a common pathophysiology (6). Patients with Ehlers-Danlos/hypermobility syndrome have widespread ECM disarray and an increase in myofibroblasts (115–117). According to the fascial armoring model these patients are expected to have a tendency to develop an imbalance in the tensegrity system because of the altered qualities of their fascia (6). In this model, an imbalanced tensegrity structure with laxity in its frame is expected to collapse more easily. The link between a collagen polymorphism and chronic widespread pain becomes more intuitive once we consider the properties of fascia.

The multifactorial interplay of fascia suggests that needling might be utilized as a method to maintain fascia in a low stiffness state. As mentioned, studies show needling lowers fascial stiffness and its shear modulus (27–29), and that myofibroblast synthesis of α-SMA is regulated by substrate rigidity (70). In disorders where fascia and tensegrity have a part in the pathophysiology, it might be useful to use TBN as a prophylaxis and maintenance. The aim would be to maintain the shear modulus of soft/myofascial tissue below the threshold of myofibroblast activity (6). Prophylaxis seems a more optimal way than trying to treat late-stage advanced disease. “Fibromyalgia,” due to high cut-off for diagnosis chosen by the American College of Rheumatology, is by definition a late-stage advanced disease likely to be more resistant to many treatment modalities and monotherapies. Since lifestyle (sedentarism, tight clothes, diet, pollution, and smoking, etc.) is suggested to be a major part of the etiology of fibromyalgia-like syndromes by inducing fascial myofibroblasts, lifestyle changes should be part of the treatment and prevention (6). Needling alone may not be enough for the cure and maintenance of remission. As long as lifestyle factors stimulate the cascade of fascial myofibroblasts, symptoms are expected to recur.

### 6.5. The problem with dry needling in light of bio-tensegrity

Because the mechanism of trigger/tender points themselves is not entirely understood, the theoretical scientific validity of western dry needling for such conditions remains just as questionable. The frequency of recurrence and possible migration of pain to different anatomical locations in the long term have not been well studied. Many studies of dry needling aim to examine the local effect of needling on a certain type of pain syndrome in a certain anatomical location, while the long-term consequences are still unclear (37, 39–41, 118–123).

It was recently suggested that trigger points and “non-specific” pains might arise due to myofibroblasts with contractile activity in the fascial bio-tensegrity system (6). These can affect nearby nerves, meaning, a painful spot is not necessarily the origin and cause of the underlying problem. Therefore, needle treatment should also be applied further away from the location of chief pain and aim to collapse the underlying tension of the bio-tensegrity system in a concentric manner. It should be performed while working according to a trigger point/satellite and myofibroblast-tensegrity-network model. The method of dry needling does not utilize these ideas, but rather “aiming and shooting at the pain” is the main strategy of this technique. According to TBN, negating one painful node in the system is not sufficient to treat the underlying problem. On the contrary: when done improperly, even if improving the local myofascial pain that it aims to treat by freeing the nerve from local tethering or tension, this false treatment might add tensions to other nodes in the structure and exacerbate the overall imbalance, and lead to other seemingly unrelated pains and symptoms. Beginning the relaxation process further away from the focus of pain while using multiple needles is sensible under this framework. It slowly and progressively starts to relax the nodes in the satellite-relationship model. In line with the bio-tensegrity model, one should recognize that the peripheries carry tension affecting the focus and should not be disregarded simply because they seem asymptomatic.

According to fascial armoring, the in-and-out maneuver stimulates local myofibroblasts to rapidly contract (*via* calcium influx and mechanosensitive stretch receptors) and leads to increased tension in the area. Increased tension leads to increase in pain. Forcefully causing edema and/or minor hemorrhage might affect fascia after multiple iterations of needle insertion. With so much pro-active destruction of fascial tissue ultimately lowering local tensions, it unfortunately neglects any conception of bio-tensegrity dynamics. Meanwhile, permitting needles to remain in fascia with patience might allow the tensegrity system to re-align according to its internal forces and release tensions in the fascial network. Rest and relaxation should allow the high-density of myofibroblasts in the network to pull and tear fascia around the needle, delicately and independently. It is also worth noting that some studies in literature have defined certain complications of treatments as “unrelated” rather than “unexplained” (124). Defining adverse events as “unrelated” may be problematic, especially if a sufficient explanation is not provided for the subjective interpretation of that phenomenon. Studies of dry needling should reflect on this fact because dry needling affects the bio-tensegrity structure, even at more distant seemingly unrelated locations.

Langevin et al. provide important evidence on acupuncture points and meridians and their relationship with connective tissue, along with evidence on the complex mechano-cellular effects of acupuncture (31, 32, 125). The effect of needling and needle manipulation on fibroblast and connective tissue is documented in studies (126, 127). As substrate rigidity and matrix mechanics deeply influence myofibroblasts and other cell types (128–132), acupuncture may be able to affect vital biomechanical, physiological, and cellular processes. The fascial connective tissue system constitutes a widespread network throughout the body, including intermuscular and subcutaneous tissue layers, and is continuous with more specialized connective tissues such as perineurium, periosteum, perimysium, peritoneum, and pleura (6, 31, 60, 85). The material surrounding blood vessels and lymphatics includes interstitial connective tissue (31). The myodural bridge connects the rectus capitis posterior with the dura (133, 134) and there is evidence that the meninges and the brain function as one network (135–138). Modulating interstitial connective tissue would cause biomechanical, vasomotor and neuromodulatory effects (31). Abnormal biotensegrity forces affecting spinal or intracranial fascia are expected not to be physiologically immaterial. Changing the fascia changes the bio-tensegrity system; and changing bio-tensegrity dynamics is expected to change the “mind” (6). The body and the mind are one being.

## 7. Conclusion

Acupuncture and TBN share several characteristics. While acupuncture may act as a global percutaneous needle fasciotomy that respects tensegrity principles, it is reasonable to suggest there are several mechanisms working in parallel in acupuncture, TBN being another mode of action in this ancient practice. Understanding the mechanism of acupuncture as tensegrity-based needling and how it can treat resistant chronic pain syndromes such as fibromyalgia may open a new avenue for medical research and practice. Examining this model in the context of other medical conditions may prove useful. In the modern era, western medicine (and humanity) aspires to follow evidence-based science; further research is needed to help reveal the mechanisms by which acupuncture targets and treats chronic non-cancer pain and functional-psychosomatic medical conditions.

## Data availability statement

The original contributions presented in this study are included in the article/supplementary material, further inquiries can be directed to the corresponding author.

## Author contributions

SP solely contributed to the study conception and design, material preparation, data collection, data curation, conceptualization, integration of information, formal analysis investigation, methodology, visualization, resources, writing—original draft, writing—review and editing, contributed to the article, and approved the submitted version.

## References

[1] Kaptchuk T. Acupuncture: theory, efficacy, and practice. *Ann Intern Med.* (2002) 136:374–83. 10.7326/0003-4819-136-5-200203050-00010 11874310

[2] Cabioðlu M Cetin B. Acupuncture and immunomodulation. *Am J Chin Med.* (2008) 36:25–36. 10.1142/S0192415X08005552 18306447

[3] Langevin H Konofagou E Badger G Churchill D Fox J Ophir J Tissue displacements during acupuncture using ultrasound elastography techniques. *Ultrasound Med Biol.* (2004) 30:1173–83. 10.1016/j.ultrasmedbio.2004.07.010 15550321

[4] Lu L Zhang Y Tang X Ge S Wen H Zeng J Evidence on acupuncture therapies is underused in clinical practice and health policy. *BMJ.* (2022) 376:e067475. 10.1136/bmj-2021-067475 35217525 PMC8868048

[5] Wen J Chen X Yang Y Liu J Li E Liu J Acupuncture medical therapy and its underlying mechanisms: a systematic review. *Am J Chin Med.* (2021) 49:1–23. 33371816 10.1142/S0192415X21500014

[6] Plaut S. Scoping review and interpretation of myofascial pain/fibromyalgia syndrome: an attempt to assemble a medical puzzle. *PLoS One.* (2022) 17:e0263087. 10.1371/journal.pone.0263087 35171940 PMC8849503

[7] Firestein G Kelley W. *Kelley’s textbook of rheumatology.* 9th Edn. Philadelphia, PA: Elsevier (2013).

[8] Wessely S Hotopf M. Is fibromyalgia a distinct clinical entity? Historical and epidemiological evidence. *Baillieres Best Pract Res Clin Rheumatol.* (1999) 13:427–36. 10.1053/berh.1999.0032 10562373

[9] Tomasek J Gabbiani G Hinz B Chaponnier C Brown R. Myofibroblasts and mechano-regulation of connective tissue remodelling. *Nat Rev Mol Cell Biol.* (2002) 3:349–63. 10.1038/nrm809 11988769

[10] Caldeira P Fonseca S Paulo A Infante J Araújo D. Linking tensegrity to sports team collective behaviors: towards the group-tensegrity hypothesis. *Sports Med Open.* (2020) 6:24. 10.1186/s40798-020-00253-y 32504195 PMC7275100

[11] Tadeo I Berbegall A Escudero L Alvaro T Noguera R. Biotensegrity of the extracellular matrix: physiology, dynamic mechanical balance, and implications in oncology and mechanotherapy. *Front Oncol.* (2014) 4:39. 10.3389/fonc.2014.00039 24624363 PMC3940942

[12] Scarr G. Biotensegrity: What is the big deal? *J Bodyw Mov Ther.* (2020) 24:134–7. 10.1016/j.jbmt.2019.09.006 31987533

[13] Wilke J Krause F Vogt L Banzer W. What is evidence-based about myofascial chains: a systematic review. *Arch Phys Med Rehabil.* (2016) 97:454–61. 10.1016/j.apmr.2015.07.023 26281953

[14] Schleip R Gabbiani G Wilke J Naylor I Hinz B Zorn A Fascia is able to actively contract and may thereby influence musculoskeletal dynamics: a histochemical and mechanographic investigation. *Front Physiol.* (2019) 10:336. 10.3389/fphys.2019.00336 31001134 PMC6455047

[15] Schleip R Klingler W. Active contractile properties of fascia. *Clin Anat.* (2019) 32:891–5. 10.1002/ca.23391 31012158

[16] Langevin H Cornbrooks C Taatjes D. Fibroblasts form a body-wide cellular network. *Histochem Cell Biol.* (2004) 122:7–15. 10.1007/s00418-004-0667-z 15221410

[17] Katz R Leavitt F Small A Small B. Intramuscular pressure is almost three times higher in fibromyalgia patients: a possible mechanism for understanding the muscle pain and tenderness. *J Rheumatol.* (2021) 48:598–602. 10.3899/jrheum.191068 32934132

[18] Kalyan-Raman U Kalyan-Raman K Yunus M Masi A. Muscle pathology in primary fibromyalgia syndrome: a light microscopic, histochemical and ultrastructural study. *J Rheumatol.* (1984) 11:808–13. 6596430

[19] Morf S Amann-Vesti B Forster A Franzeck U Koppensteiner R Uebelhart D Microcirculation abnormalities in patients with fibromyalgia – measured by capillary microscopy and laser fluxmetry. *Arthritis Res Ther.* (2005) 7:R209–16. 10.1186/ar1459 15743467 PMC1065312

[20] Sprott H Salemi S Gay R Bradley L Alarcón G Oh S Increased DNA fragmentation and ultrastructural changes in fibromyalgic muscle fibres. *Ann Rheum Dis.* (2004) 63:245–51. 10.1136/ard.2002.004762 14962957 PMC1754917

[21] Evdokimov D Kreß L Dinkel P Frank J Sommer C Üçeyler N. Pain-associated mediators and axon pathfinders in fibromyalgia skin cells. *J Rheumatol.* (2020) 47:140–8. 10.3899/jrheum.190248 31203217

[22] Ramírez-Tejero J Martínez-Lara E Peinado MÁ Del Moral ML Siles E. Hydroxytyrosol as a promising ally in the treatment of fibromyalgia. *Nutrients.* (2020) 12:2386. 10.3390/nu12082386 32784915 PMC7468876

[23] Gronemann S Ribel-Madsen S Bartels E Danneskiold-Samsoe B Bliddal H. Collagen and muscle pathology in fibromyalgia patients. *Rheumatology.* (2004) 43:27–31. 10.1093/rheumatology/keg452 12867573

[24] Cummings T White A. Needling therapies in the management of myofascial trigger point pain: a systematic review. *Arch Phys Med Rehabil.* (2001) 82:986–92. 10.1053/apmr.2001.24023 11441390

[25] Frost F Jessen B Siggaard-Andersen J. A control, double-blind comparison of mepivacaine injection versus saline injection for myofascial pain. *Lancet.* (1980) 1:499–500. 10.1016/s0140-6736(80)92761-0 6102230

[26] Lewit K. The needle effect in the relief of myofascial pain. *Pain.* (1979) 6:83–90. 10.1016/0304-3959(79)90142-8 424236

[27] Luan S Zhu Z Ruan J Lin C Ke S Xin W Randomized trial on comparison of the efficacy of extracorporeal shock wave therapy and dry needling in myofascial trigger points. *Am J Phys Med Rehabil.* (2019) 98:677–84. 10.1097/PHM.0000000000001173 31318748

[28] Maher R Hayes D Shinohara M. Quantification of dry needling and posture effects on myofascial trigger points using ultrasound shear-wave elastography. *Arch Phys Med Rehabil.* (2013) 94:2146–50. 10.1016/j.apmr.2013.04.021 23684553

[29] Sánchez-Infante J Bravo-Sánchez A Jiménez F Abián-Vicén J. Effects of dry needling on muscle stiffness in latent myofascial trigger points: a randomized controlled trial. *J Pain.* (2021) 22:817–25. 10.1016/j.jpain.2021.02.004 33636373

[30] Fox J Gray W Koptiuch C Badger G Langevin H. Anisotropic tissue motion induced by acupuncture needling along intermuscular connective tissue planes. *J Altern Complement Med.* (2014) 20:290–4. 10.1089/acm.2013.0397 24593827 PMC3995146

[31] Langevin H Churchill D Cipolla M. Mechanical signaling through connective tissue: a mechanism for the therapeutic effect of acupuncture. *FASEB J.* (2001) 15:2275–82. 10.1096/fj.01-0015hyp 11641255

[32] Langevin H Churchill D Fox J Badger G Garra B Krag M. Biomechanical response to acupuncture needling in humans. *J Appl Physiol.* (2001) 91:2471–8. 10.1152/jappl.2001.91.6.2471 11717207

[33] Tomasek J Haaksma C Eddy R Vaughan M. Fibroblast contraction occurs on release of tension in attached collagen lattices: dependency on an organized actin cytoskeleton and serum. *Anat Rec.* (1992) 232:359–68. 10.1002/ar.1092320305 1543260

[34] Deare J Zheng Z Xue C Liu J Shang J Scott S Acupuncture for treating fibromyalgia. *Cochrane Database Syst Rev.* (2013) 2013:CD007070. 10.1002/14651858.CD007070.pub2 23728665 PMC4105202

[35] Furlan A van Tulder M Cherkin D Tsukayama H Lao L Koes B Acupuncture and dry-needling for low back pain. *Cochrane Database Syst Rev.* (2005) 1:CD001351. 10.1002/14651858.CD001351.pub2 15674876 PMC12145953

[36] Li X Wang R Xing X Shi X Tian J Zhang J Acupuncture for myofascial pain syndrome: a network meta-analysis of 33 randomized controlled trials. *Pain Physician.* (2017) 20:E883–902. 28934793

[37] Liu L Huang Q Liu Q Thitham N Li L Ma Y Evidence for dry needling in the management of myofascial trigger points associated with low back pain: a systematic review and meta-analysis. *Arch Phys Med Rehabil.* (2018) 99:144–52.e2. 10.1016/j.apmr.2017.06.008 28690077

[38] Zhang X Chen H Xu W Song Y Gu Y Ni G. Acupuncture therapy for fibromyalgia: a systematic review and meta-analysis of randomized controlled trials. *J Pain Res.* (2019) 12:527–42. 10.2147/JPR.S186227 30787631 PMC6365227

[39] Gattie E Cleland J Snodgrass S. The effectiveness of trigger point dry needling for musculoskeletal conditions by physical therapists: a systematic review and meta-analysis. *J Orthop Sports Phys Ther.* (2017) 47:133–49. 10.2519/jospt.2017.7096 28158962

[40] Liu L Huang Q Liu Q Ye G Bo C Chen M Effectiveness of dry needling for myofascial trigger points associated with neck and shoulder pain: a systematic review and meta-analysis. *Arch Phys Med Rehabil.* (2015) 96:944–55. 10.1016/j.apmr.2014.12.015 25576642

[41] Hu H Gao H Ma R Zhao X Tian H Li L. Is dry needling effective for low back pain?: A systematic review and PRISMA-compliant meta-analysis. *Medicine.* (2018) 97:e11225. 10.1097/MD.0000000000011225 29952980 PMC6242300

[42] De Meulemeester K Calders P Cagnie B. Exploring the underlying mechanisms of action of dry needling: what is the immediate effect on muscle electrophysiology? An experimental randomized controlled trial. *Am J Phys Med Rehabil.* (2022) 101:18–25. 10.1097/PHM.0000000000001732 34915542

[43] Hsieh Y Chou L Joe Y Hong C. Spinal cord mechanism involving the remote effects of dry needling on the irritability of myofascial trigger spots in rabbit skeletal muscle. *Arch Phys Med Rehabil.* (2011) 92:1098–105. 10.1016/j.apmr.2010.11.018 21529778

[44] Perreault T Fernández-de-Las-Peñas C Cummings M Gendron B. Needling interventions for sciatica: choosing methods based on neuropathic pain mechanisms-a scoping review. *J Clin Med.* (2021) 10:2189. 10.3390/jcm10102189 34069357 PMC8158699

[45] Rajkannan P Vijayaraghavan R. Dry needling in chronic abdominal wall pain of uncertain origin. *J Bodyw Mov Ther.* (2019) 23:94–8. 10.1016/j.jbmt.2018.01.004 30691770

[46] Martín-Pintado-Zugasti A Mayoral Del Moral O Gerwin R Fernández-Carnero J. Post-needling soreness after myofascial trigger point dry needling: current status and future research. *J Bodyw Mov Ther.* (2018) 22:941–6. 10.1016/j.jbmt.2018.01.003 30368339

[47] Lumley M Schubiner H Lockhart N Kidwell K Harte S Clauw D Emotional awareness and expression therapy, cognitive behavioral therapy, and education for fibromyalgia: a cluster-randomized controlled trial. *Pain.* (2017) 158:2354–63. 10.1097/j.pain.0000000000001036 28796118 PMC5680092

[48] Sluka K Clauw D. Neurobiology of fibromyalgia and chronic widespread pain. *Neuroscience.* (2016) 338:114–29. 10.1016/j.neuroscience.2016.06.006 27291641 PMC5083139

[49] Linde K Allais G Brinkhaus B Fei Y Mehring M Shin B Acupuncture for the prevention of tension-type headache. *Cochrane Database Syst Rev.* (2016) 4:CD007587. 10.1002/14651858.CD007587.pub2 27092807 PMC4955729

[50] Linde K Allais G Brinkhaus B Fei Y Mehring M Vertosick E Acupuncture for the prevention of episodic migraine. *Cochrane Database Syst Rev.* (2016) 2016:CD001218. 10.1002/14651858.CD001218.pub3 27351677 PMC4977344

[51] Vickers A Vertosick E Lewith G MacPherson H Foster N Sherman K Acupuncture for chronic pain: update of an individual patient data meta-analysis. *J Pain.* (2018) 19:455–74. 10.1016/j.jpain.2017.11.005 29198932 PMC5927830

[52] Smith C Armour M Lee M Wang L Hay P. Acupuncture for depression. *Cochrane Database Syst Rev.* (2018) 3:CD004046. 10.1002/14651858.CD004046.pub4 29502347 PMC6494180

[53] Yuan Q Wang P Liu L Sun F Cai Y Wu W Acupuncture for musculoskeletal pain: a meta-analysis and meta-regression of sham-controlled randomized clinical trials. *Sci Rep.* (2016) 6:30675. 10.1038/srep30675 27471137 PMC4965798

[54] Vincent C. The safety of acupuncture. *BMJ.* (2001) 323:467–8. 10.1136/bmj.323.7311.467 11532826 PMC1121068

[55] Yamashita H Tsukayama H White A Tanno Y Sugishita C Ernst E. Systematic review of adverse events following acupuncture: the Japanese literature. *Complement Ther Med.* (2001) 9:98–104. 10.1054/ctim.2001.0446 11444889

[56] Bensoussan A Myers S Carlton A. Risks associated with the practice of traditional Chinese medicine: an Australian study. *Arch Fam Med.* (2000) 9:1071–8. 10.1001/archfami.9.10.1071 11115210

[57] Ernst E White A. Life-threatening adverse reactions after acupuncture? A systematic review. *Pain.* (1997) 71:123–6. 10.1016/s0304-3959(97)03368-x 9211472

[58] Yamashita H Tsukayama H Tanno Y Nishijo K. Adverse events related to acupuncture. *JAMA.* (1998) 280:1563–4. 10.1001/jama.280.18.1563-a 9820249

[59] Williams P Goldspink G. Connective tissue changes in immobilised muscle. *J Anat.* (1984) 138:343–50. 6715254 PMC1164074

[60] Wilke J Schleip R Yucesoy C Banzer W. Not merely a protective packing organ? A review of fascia and its force transmission capacity. *J Appl Physiol.* (2018) 124:234–44. 10.1152/japplphysiol.00565.2017 29122963

[61] Li A Liu Y Plott J Chen L Montgomery J Shih A. Multi-bevel needle design enabling accurate insertion in biopsy for cancer diagnosis. *IEEE Trans Biomed Eng.* (2021) 68:1477–86. 10.1109/TBME.2021.3054922 33507862 PMC8104469

[62] Li A Putra K Chen L Montgomery J Shih A. Mosquito proboscis-inspired needle insertion to reduce tissue deformation and organ displacement. *Sci Rep.* (2020) 10:12248. 10.1038/s41598-020-68596-w 32699296 PMC7376018

[63] Langevin H Churchill D Wu J Badger G Yandow J Fox J Evidence of connective tissue involvement in acupuncture. *FASEB J.* (2002) 16:872–4. 10.1096/fj.01-0925fje 11967233

[64] Ernberg M Hedenberg-Magnusson B Alstergren P Kopp S. The level of serotonin in the superficial masseter muscle in relation to local pain and allodynia. *Life Sci.* (1999) 65:313–25. 10.1016/s0024-3205(99)00250-7 10447217

[65] Moraska A Hickner R Kohrt W Brewer A. Changes in blood flow and cellular metabolism at a myofascial trigger point with trigger point release (ischemic compression): a proof-of-principle pilot study. *Arch Phys Med Rehabil.* (2013) 94:196–200. 10.1016/j.apmr.2012.08.216 22975226 PMC3529849

[66] Shah J Gilliams E. Uncovering the biochemical milieu of myofascial trigger points using in vivo microdialysis: an application of muscle pain concepts to myofascial pain syndrome. *J Bodyw Mov Ther.* (2008) 12:371–84. 10.1016/j.jbmt.2008.06.006 19083696

[67] Gerdle B Söderberg K Salvador Puigvert L Rosendal L Larsson B. Increased interstitial concentrations of pyruvate and lactate in the trapezius muscle of patients with fibromyalgia: a microdialysis study. *J Rehabil Med.* (2010) 42:679–87. 10.2340/16501977-0581 20603699

[68] Dong Z Shun-Yue L Shu-You W Hui-Min M. Evaluation of influence of acupuncture and electro-acupuncture for blood perfusion of stomach by laser Doppler blood perfusion imaging. *Evid Based Complement Alternat Med.* (2011) 2011:969231. 10.1093/ecam/nep050 19531626 PMC3137869

[69] Dorigo B Bartoli V Grisillo D Beconi D. Fibrositic myofascial pain in intermittent claudication. Effect of anesthetic block of trigger points on exercise tolerance. *Pain.* (1979) 6:183–90. 10.1016/0304-3959(79)90125-8 287998

[70] Goffin J Pittet P Csucs G Lussi J Meister J Hinz B. Focal adhesion size controls tension-dependent recruitment of alpha-smooth muscle actin to stress fibers. *J Cell Biol.* (2006) 172:259–68. 10.1083/jcb.200506179 16401722 PMC2063555

[71] Kural M Billiar K. Myofibroblast persistence with real-time changes in boundary stiffness. *Acta Biomater.* (2016) 32:223–30. 10.1016/j.actbio.2015.12.031 26712600 PMC4754153

[72] Moog P Buchner L Cerny M Schmauss D Megerle K Erne H. Analysis of recurrence and complications after percutaneous needle fasciotomy in Dupuytren’s disease. *Arch Orthop Trauma Surg.* (2019) 139:1471–7. 10.1007/s00402-019-03247-y 31367843

[73] Soreide E Murad M Denbeigh J Lewallen E Dudakovic A Nordsletten L Treatment of Dupuytren’s contracture: a systematic review. *Bone Joint J.* (2018) 100-B:1138–45. 10.1302/0301-620X.100B9.BJJ-2017-1194.R2 30168768

[74] Balfour W. Observations on the pathology and cure of rheumatism. *Edinb Med Surg J.* (1815) 11:168–87. PMC5743373 30329434

[75] Hopwood V. *Acupuncture in physiotherapy: Key concepts and evidence-based practice.* Oxford: Elsevier Butterworth-Heinemann (2004).

[76] Tan R Rush S. *Twelve and twelve in acupuncture: advanced principles and techniques.* San Diego, CA: Richard Tan Publishing (1991).

[77] Guo Q Di Z Tian H Zhang Q. Contralateral acupuncture for the treatment of phantom limb pain and phantom limb sensation in oncologic lower limb amputee: a case report. *Front Neurosci.* (2021) 15:713548. 10.3389/fnins.2021.713548 34744604 PMC8568952

[78] Karagounis P Tsironi M Prionas G Tsiganos G Baltopoulos P. Treatment of plantar fasciitis in recreational athletes: two different therapeutic protocols. *Foot Ankle Spec.* (2011) 4:226–34. 10.1177/1938640011407320 21868796

[79] Wang W Liu Y Jiao R Liu S Zhao J Liu Z. Comparison of electroacupuncture and manual acupuncture for patients with plantar heel pain syndrome: a randomized controlled trial. *Acupunct Med.* (2021) 39:272–82. 10.1177/0964528420947739 32811186

[80] Harty J Soffe K O’Toole G Stephens M. The role of hamstring tightness in plantar fasciitis. *Foot Ankle Int.* (2005) 26:1089–92. 10.1177/107110070502601215 16390645

[81] Labovitz J Yu J Kim C. The role of hamstring tightness in plantar fasciitis. *Foot Ankle Spec.* (2011) 4:141–4. 10.1177/1938640010397341 21368068

[82] Shin K Kim J Lee S Shin M Kim T Park H Acupuncture for lateral epicondylitis (tennis elbow): study protocol for a randomized, practitioner-assessor blinded, controlled pilot clinical trial. *Trials.* (2013) 14:174. 10.1186/1745-6215-14-174 23768129 PMC3685553

[83] Melchart D Streng A Hoppe A Brinkhaus B Witt C Wagenpfeil S Acupuncture in patients with tension-type headache: randomised controlled trial. *BMJ.* (2005) 331:376–82. 10.1136/bmj.38512.405440.8F 16055451 PMC1184247

[84] Nordez A Gross R Andrade R Le Sant G Freitas S Ellis R Non-muscular structures can limit the maximal joint range of motion during stretching. *Sports Med.* (2017) 47:1925–9. 10.1007/s40279-017-0703-5 28255938

[85] Stecco A Macchi V Stecco C Porzionato A Ann Day J Delmas V Anatomical study of myofascial continuity in the anterior region of the upper limb. *J Bodyw Mov Ther.* (2009) 13:53–62. 10.1016/j.jbmt.2007.04.009 19118793

[86] Wilke J Krause F. Myofascial chains of the upper limb: a systematic review of anatomical studies. *Clin Anat.* (2019) 32:934–40. 10.1002/ca.23424 31226229

[87] Prabu Raja G Shyamasunder Bhat N Marie Cruz A Prabhu A Fernandes S Naaz N. The anatomical myofascial continuum between the neck and eyes. *Clin Anat.* (2022) 35:340–6. 10.1002/ca.23835 35043988

[88] Focks C März U Hosbach I. *Atlas of acupuncture.* Edinburgh: Churchill Livingstone/Elsevier (2008).

[89] Veith I. Huang Ti Nei ching Su Wên: The Yellow Emperor’s classic of internal medicine. Chapters 1-34 translated from the Chinese, with an introductory study by Ilza Veith. Berkeley, CA: University of California Press (1972).

[90] Yang Z Liu L. *The Yellow Emperor’s Inner Transmission of Acupuncture.* Hong Kong: The Chinese University of Hong Kong Press (2020).

[91] Melzack R Stillwell D Fox E. Trigger points and acupuncture points for pain: correlations and implications. *Pain.* (1977) 3:3–23. 10.1016/0304-3959(77)90032-X 69288

[92] Sasabe R Sakamoto J Goto K Honda Y Kataoka H Nakano J Effects of joint immobilization on changes in myofibroblasts and collagen in the rat knee contracture model. *J Orthop Res.* (2017) 5:1998–2006. 10.1002/jor.23498 27918117

[93] Wan R Weissman J Grundman K Lang L Grybowski D Galiano R. Diabetic wound healing: The impact of diabetes on myofibroblast activity and its potential therapeutic treatments. *Wound Repair Regen.* (2021) 29:573–81. 10.1111/wrr.12954 34157786

[94] Wolfson B Zhang Y Gernapudi R Duru N Yao Y Lo P A high-fat diet promotes mammary gland myofibroblast differentiation through MicroRNA 140 downregulation. *Mol Cell Biol.* (2017) 37:e00461–16. 10.1128/MCB.00461-16 27895151 PMC5288574

[95] Yang W Zhang S Zhu J Jiang H Jia D Ou T Gut microbe-derived metabolite trimethylamine N-oxide accelerates fibroblast-myofibroblast differentiation and induces cardiac fibrosis. *J Mol Cell Cardiol.* (2019) 134:119–30. 10.1016/j.yjmcc.2019.07.004 31299216

[96] Arora P Bibby K McCulloch C. Slow oscillations of free intracellular calcium ion concentration in human fibroblasts responding to mechanical stretch. *J Cell Physiol.* (1994) 161:187–200. 10.1002/jcp.1041610202 7962103

[97] Castella L Buscemi L Godbout C Meister J Hinz B. A new lock-step mechanism of matrix remodelling based on subcellular contractile events. *J Cell Sci.* (2010) 123:1751–60. 10.1242/jcs.066795 20427321

[98] Kluger B Rakowski D Christian M Cedar D Wong B Crawford J Randomized, controlled trial of acupuncture for fatigue in parkinson’s disease. *Mov Disord.* (2016) 31:1027–32. 10.1002/mds.26597 27028133

[99] Mist S Jones K. Randomized controlled trial of acupuncture for women with fibromyalgia: group acupuncture with traditional chinese medicine diagnosis-based point selection. *Pain Med.* (2018) 19:1862–71. 10.1093/pm/pnx322 29447382 PMC6127237

[100] Wu L. Nonlinear finite element analysis for musculoskeletal biomechanics of medial and lateral plantar longitudinal arch of Virtual Chinese Human after plantar ligamentous structure failures. *Clin Biomech.* (2007) 22:221–9. 10.1016/j.clinbiomech.2006.09.009 17118500

[101] Padhi A Singh K Franco-Barraza J Marston D Cukierman E Hahn K Force-exerting perpendicular lateral protrusions in fibroblastic cell contraction. *Commun Biol.* (2020) 3:390. 10.1038/s42003-020-01117-7 32694539 PMC7374753

[102] Verjee L Midwood K Davidson D Essex D Sandison A Nanchahal J. Myofibroblast distribution in Dupuytren’s cords: correlation with digital contracture. *J Hand Surg Am.* (2009) 34:1785–94. 10.1016/j.jhsa.2009.08.005 19910144

[103] VandeBerg J Rudolph R Gelberman R Woodward M. Ultrastructural relationship of skin to nodule and cord in Dupuytren’s contracture. *Plast Reconstr Surg.* (1982) 69:835–44. 10.1097/00006534-198205000-00021 7071229

[104] Bisson M McGrouther D Mudera V Grobbelaar A. The different characteristics of Dupuytren’s disease fibroblasts derived from either nodule or cord: expression of alpha-smooth muscle actin and the response to stimulation by TGF-beta1. *J Hand Surg Br.* (2003) 28:351–6. 10.1016/s0266-7681(03)00135-9 12849947

[105] Ng C Hinz B Swartz M. Interstitial fluid flow induces myofibroblast differentiation and collagen alignment in vitro. *J Cell Sci.* (2005) 118:4731–9. 10.1242/jcs.02605 16188933

[106] Ma H Macdougall L GonzalezRodriguez A Schroeder M Batan D Weiss R Calcium signaling regulates valvular interstitial cell alignment and myofibroblast activation in fast-relaxing boronate hydrogels. *Macromol Biosci.* (2020) 20:e2000268. 10.1002/mabi.202000268 32924320 PMC7773027

[107] Soiné J Hersch N Dreissen G Hampe N Hoffmann B Merkel R Measuring cellular traction forces on non-planar substrates. *Interface Focus.* (2016) 6:20160024. 10.1098/rsfs.2016.0024 27708757 PMC4992736

[108] Cirka H Monterosso M Diamantides N Favreau J Wen Q Billiar K. Active traction force response to long-term cyclic stretch is dependent on cell pre-stress. *Biophys J.* (2016) 110:1845–57. 10.1016/j.bpj.2016.02.036 27119644 PMC4850240

[109] Vassaux M Milan J. Stem cell mechanical behaviour modelling: substrate’s curvature influence during adhesion. *Biomech Model Mechanobiol.* (2017) 16:1295–308. 10.1007/s10237-017-0888-4 28224241 PMC5511597

[110] Staud R. Peripheral pain mechanisms in chronic widespread pain. *Best Pract Res Clin Rheumatol.* (2011) 25:155–64. 10.1016/j.berh.2010.01.010 22094192 PMC3220877

[111] Acasuso-Díaz M Collantes-Estévez E. Joint hypermobility in patients with fibromyalgia syndrome. *Arthritis Care Res.* (1998) 11:39–42. 10.1002/art.1790110107 9534492

[112] Sacheti A Szemere J Bernstein B Tafas T Schechter N Tsipouras P. Chronic pain is a manifestation of the Ehlers-Danlos syndrome. *J Pain Symptom Manage.* (1997) 14:88–93. 10.1016/s0885-3924(97)00007-9 9262038

[113] Gedalia A Press J Klein M Buskila D. Joint hypermobility and fibromyalgia in schoolchildren. *Ann Rheum Dis.* (1993) 52:494–6. 10.1136/ard.52.7.494 8346976 PMC1005086

[114] Chen G Olver J Kanaan R. Functional somatic syndromes and joint hypermobility: a systematic review and meta-analysis. *J Psychosom Res.* (2021) 148:110556. 10.1016/j.jpsychores.2021.110556 34237584

[115] Chiarelli N Zoppi N Ritelli M Venturini M Capitanio D Gelfi C Biological insights in the pathogenesis of hypermobile Ehlers-Danlos syndrome from proteome profiling of patients’ dermal myofibroblasts. *Biochim Biophys Acta Mol Basis Dis.* (2021) 1867:166051. 10.1016/j.bbadis.2020.166051 33383104

[116] Zoppi N Chiarelli N Binetti S Ritelli M Colombi M. Dermal fibroblast-to-myofibroblast transition sustained by αvß3 integrin-ILK-Snail1/Slug signaling is a common feature for hypermobile Ehlers-Danlos syndrome and hypermobility spectrum disorders. *Biochim Biophys Acta Mol Basis Dis.* (2018) 1864:1010–23. 10.1016/j.bbadis.2018.01.005 29309923

[117] Chiarelli N Zoppi N Venturini M Capitanio D Gelfi C Ritelli M Matrix metalloproteinases inhibition by doxycycline rescues extracellular matrix organization and partly reverts myofibroblast differentiation in hypermobile ehlers-danlos syndrome dermal fibroblasts: a potential therapeutic target? *Cells.* (2021) 10:3236. 10.3390/cells10113236 34831458 PMC8621259

[118] Llamas-Ramos R Pecos-Martín D Gallego-Izquierdo T Llamas-Ramos I Plaza-Manzano G Ortega-Santiago R Comparison of the short-term outcomes between trigger point dry needling and trigger point manual therapy for the management of chronic mechanical neck pain: a randomized clinical trial. *J Orthop Sports Phys Ther.* (2014) 44:852–61. 10.2519/jospt.2014.5229 25269764

[119] Mejuto-Vázquez M Salom-Moreno J Ortega-Santiago R Truyols-Domínguez S Fernández-de-Las-Peñas C. Short-term changes in neck pain, widespread pressure pain sensitivity, and cervical range of motion after the application of trigger point dry needling in patients with acute mechanical neck pain: a randomized clinical trial. *J Orthop Sports Phys Ther.* (2014) 44:252–60. 10.2519/jospt.2014.5108 24568260

[120] Calvo-Lobo C Pacheco-da-Costa S Martínez-Martínez J Rodríguez-Sanz D Cuesta-Álvaro P López-López D. Dry needling on the infraspinatus latent and active myofascial trigger points in older adults with nonspecific shoulder pain: a randomized clinical trial. *J Geriatr Phys Ther.* (2018) 41:1–13. 10.1519/JPT.0000000000000079 26760574 PMC5728593

[121] Geist K Frierson E Goudiss H Kitchen H Wilkins M Pruszynski D Short-term effects of dry needling at a spinal and peripheral site on functional outcome measures, strength, and proprioception among individuals with a lateral ankle sprain. *J Bodyw Mov Ther.* (2021) 26:158–66. 10.1016/j.jbmt.2020.12.021 33992238

[122] Kalia V Mani S Kumar S. Short-term effect of myofascial trigger point dry-needling in patients with Adhesive Capsulitis. *J Bodyw Mov Ther.* (2021) 25:146–50. 10.1016/j.jbmt.2020.10.014 33714486

[123] Fernández-De-Las-Peñas C Plaza-Manzano G Sanchez-Infante J Gómez-Chiguano G Cleland J Arias-Buría J Is dry needling effective when combined with other therapies for myofascial trigger points associated with neck pain symptoms? A systematic review and meta-analysis. *Pain Res Manag.* (2021) 2021:8836427. 10.1155/2021/8836427 33603940 PMC7872772

[124] Mishra A Skrepnik N Edwards S Jones G Sampson S Vermillion D Efficacy of platelet-rich plasma for chronic tennis elbow: a double-blind, prospective, multicenter, randomized controlled trial of 230 patients. *Am J Sports Med.* (2014) 42:463–71. 10.1177/0363546513494359 23825183

[125] Langevin H Yandow J. Relationship of acupuncture points and meridians to connective tissue planes. *Anat Rec.* (2002) 269:257–65. 10.1002/ar.10185 12467083

[126] Langevin H Bouffard N Churchill D Badger G. Connective tissue fibroblast response to acupuncture: dose-dependent effect of bidirectional needle rotation. *J Altern Complement Med.* (2007) 13:355–60. 10.1089/acm.2007.6351 17480137 PMC3065718

[127] Langevin H Bouffard N Badger G Churchill D Howe A. Subcutaneous tissue fibroblast cytoskeletal remodeling induced by acupuncture: evidence for a mechanotransduction-based mechanism. *J Cell Physiol.* (2006) 207:767–74. 10.1002/jcp.20623 16511830

[128] Kayal C Moeendarbary E Shipley R Phillips J. Mechanical response of neural cells to physiologically relevant stiffness gradients. *Adv Healthc Mater.* (2020) 9:e1901036. 10.1002/adhm.201901036 31793251 PMC8407326

[129] Gu Y Ji Y Zhao Y Liu Y Ding F Gu X The influence of substrate stiffness on the behavior and functions of Schwann cells in culture. *Biomaterials.* (2012) 33:6672–81. 10.1016/j.biomaterials.2012.06.006 22738780

[130] Yip A Iwasaki K Ursekar C Machiyama H Saxena M Chen H Cellular response to substrate rigidity is governed by either stress or strain. *Biophys J.* (2013) 104:19–29. 10.1016/j.bpj.2012.11.3805 23332055 PMC3540269

[131] Doss B Pan M Gupta M Grenci G Mège R Lim C Cell response to substrate rigidity is regulated by active and passive cytoskeletal stress. *Proc Natl Acad Sci U.S.A.* (2020) 117:12817–25. 10.1073/pnas.1917555117 32444491 PMC7293595

[132] Discher D Janmey P Wang Y. Tissue cells feel and respond to the stiffness of their substrate. *Science.* (2005) 310:1139–43. 10.1126/science.1116995 16293750

[133] Zheng N Yuan X Chi Y Liu P Wang B Sui J The universal existence of myodural bridge in mammals: an indication of a necessary function. *Sci Rep.* (2017) 7:8248. 10.1038/s41598-017-06863-z 28811472 PMC5557938

[134] Jiang W Zhang Z Yu S Sun J Ding S Ma G Scanning electron microscopic observation of myodural bridge in the human suboccipital region. *Spine.* (2020) 45:E1296–301. 10.1097/BRS.0000000000003602 32796464

[135] Bose A Basu R Maulik M Das Sarma J. Loss of Cx43-mediated functional gap junction communication in meningeal fibroblasts following mouse hepatitis virus infection. *Mol Neurobiol.* (2018) 55:6558–71. 10.1007/s12035-017-0861-3 29327203 PMC7090783

[136] Grafstein B Liu S Cotrina M Goldman S Nedergaard M. Meningeal cells can communicate with astrocytes by calcium signaling. *Ann Neurol.* (2000) 47:18–25. 10632097

[137] Mercier F Hatton G. Immunocytochemical basis for a meningeo-glial network. *J Comp Neurol.* (2000) 420:445–65. 10.1002/(sici)1096-9861(20000515)420:4<445::aid-cne4>3.0.co;2-3 10805920

[138] Mercier F Hatton G. Connexin 26 and basic fibroblast growth factor are expressed primarily in the subpial and subependymal layers in adult brain parenchyma: roles in stem cell proliferation and morphological plasticity? *J Comp Neurol.* (2001) 431:88–104. 10.1002/1096-9861(20010226)431:1<88::aid-cne1057>3.0.co;2-d 11169992

